# Response to: DNA identification by pedigree likelihood ratio accommodating population substructure and mutations- authors' reply

**DOI:** 10.1186/2041-2223-2-8

**Published:** 2011-03-25

**Authors:** Ranajit Chakraborty, Jianye Ge, Bruce Budowle

**Affiliations:** 1 Department of Forensic and Investigative Genetics, University of North Texas Health Science Center, Fort Worth, Texas 76107, USA; 2 Institute of Investigative Genetics, University of North Texas Health Science Center, Fort Worth, Texas 76107, USA

## 

Dear Editor,

In their letter to the editor, Egeland *et al*. [1] criticize the mutation model used in our paper [2], and propose that our comments about the mutation model used by Dawid *et al*. [3] are not convincing, because we do not provide any data in support of our assertions. Their criticisms are primarily based on three premises: 1) that our mutation model, presented on page 5 of our paper [2], is mathematically incorrect, because our equation 8 does not define a proper probability distribution (that is, the probabilities do not add to 1); 2) that our mutation model allows for production of alleles of zero or negative repeat sizes, which are not meaningful; and 3) that the model used in the paper by Dawid *et al *[3] uses the relationship between mutational transition probabilities and allele frequency on the basis that allele frequencies are representative of the stationary distribution of a mutation process, and hence, in the absence of natural selection, is presumably applicable to the sequence tagged repeat (STR) loci used in DNA forensics. Each of these issues needs further discussion, and we thank the authors for giving us an opportunity to explain them further.

First, the mutation model, explained by equation 8 of page 5 of our paper [2], clearly states that the geometric distribution for Pr (*X *= *x*) applies to 'alleles to change by adding or subtracting an absolute number of x repeat units'. Hence, by definition *x *> 0, and as noted just after equation 8 'equal probabilities for gaining or losing repeats are assumed', it is incorrect to multiply the geometric terms by a factor of 2, as Egeland *et al. have done *[1]. Following this logic, our equation 8 mathematically represents a valid probability distribution, because the total probability of mutation (that is, *X *≠ 0) becomes μ, by summing the individual terms over all non-zero positive integer values of *X*. In addition, we are not the first to use such formulations of a mutation model. Estoup *et al*. [4] used exactly the same representation for the two-phase mutation model of Di Rienzo *et al*. [5] (see the information box 1 on page 1592 of the report by Estoup *et al*. [4]). Both of these papers provide observational and theoretical support for such a mutation model, applicable to STR loci (microsatellites, in their terminology).

Second, it is true that this mutation model allows for production of zero or negative repeat sizes of alleles. This is also true for the simple stepwise mutation model (SMM [6]), in which, by successive single-step changes towards contraction of allele sizes, the allelic states (designated by repeat size) can eventually become zero or negative. There are alternative methods to minimize the effect of such biological absurdities. For example, allele-size constraints may be introduced to avoid continual unlimited expansions or contractions [7,8], although evidence for the presence of such constraints for STRs without any phenotypic effect is not clearly established [8]. By contrast, near equiprobable contraction and expansion, together with decreased probability of large multistep mutations, reduce the chance for reaching absurd allele sizes to almost negligible values (discussed below). An alternative method is to add allele-size nomenclatures denoted by <*a *or >*b*, for some arbitrary allele sizes *a *and *b*, defined by the smallest and largest sizes of alleles found at a locus in worldwide surveys (often called 'below and above allelic ladder alleles' [9-11]). The example chosen by Egeland *et al*. [1] also overemphasizes the possible occurrences of such unrealistic mutations. Incorrectly citing us, they used a mutation rate of 10^-3 ^for the TH01 locus whereas in our paper, we only said that the STR loci used in forensic DNA analyses have mutation rates in the order of 10^-3 ^to 10^-4^/locus/generation. Compilations of mutational data in the AABB annual reports [12] suggest that TH01is the least mutable of the forensic STR loci, with an overall rate of mutation (μ) of the order of 10^-4^/locus/generation. Thus, even if all mutations at the TH01 locus occur only from the allele of repeat size 3 (which obviously cannot be the case), with α = 0.95 and μ = 10^-4^, the chances of zero or negative allele sizes occurring by mutation is 1.25 × 10^-7^, an order of magnitude smaller than that suggested by Egeland *et al*. [1]. In fact, numerical results of their own mutation model, as described on page 57 of the report by Dawid *et al*. [3], neglect mutational possibilities that may be more frequent than this, which we considered 'negligible'.

Third, Egeland *et al*. [1] claim that our mutation model, together with several others listed on page 59 of the report by Dawid *et al*. [3], does not lead to a stationary distribution of allele frequencies. In fact, their notion of stationary allele frequency is biologically untenable for evolutionary genetic models of mutations. By definition, mutation is the basic evolutionary mechanism by which new variants are introduced into a population, and hence, the frequencies of any prescribed given allele (designated by repeat size) cannot have a stationary state under the balance of mutation and other evolutionary factors (such as genetic drift in a finite population). Further, it has been known for almost four decades that when alleles are labeled by specific allelic states (such as repeat sizes for STR loci), under a mutation-drift balance, frequencies of alleles of specific designations always fluctuate over time (called 'wandering distributions' by Moran [13]), and hence, there is no equilibrium frequency of alleles unless there is a fixed finite set of possible alleles among which mutational transitions occur [6,14]. Because there is no pre-assigned fixed set of allelic states for the most mechanisms of mutations at STR loci (such as replication slippage or non-homologous recombination), the concept of a fixed finite set of STR alleles is biologically unrealistic. However, stationary distributions of allelic diversity (called allele frequency spectra) at such loci have been described and theoretically shown to exist under the infinite allele model [14] and the single-step and multistep mutation models [6,15]. The allele frequency spectrum, in such formulations, is defined by the number of copies of alleles with any assigned frequency range of alleles, denoted as *φ(x)dx*, meaning that there are *φ(x) *many alleles with allele frequency in the range of (*x, x + dx*). However, these allelic states have a 'wandering' nature, because of the continual new introduction of mutations. Published research starting from the early 1990 s and continuing to the present day support some form of a generalized stepwise mutation model as an approximation of the governing mutation model for STR loci [4,5,7,8,16-18]. By contrast, Egeland *et al*. [1] gave citations of a minisatellite study [19] and some expert systems [20,21] as supporting evidence for their mutation model [3], without recognizing that the evolutionary processes underlying minisatellite loci are considerably different from those underlying STR loci, particularly those used in DNA forensics, and expert systems are not true validation of the biological processes underlying such mutations.

Apart from such statistical support of stepwise mutation models for STR polymorphisms, there is also empirical support for the occurrence of such mutations, as seen from compilations of experiences of mutational observations in parentage testing laboratories [12]. Such data show that, although most mutations (about 95%) involve single-step contraction or expansion (almost in equal proportions), occasional multistep changes are also seen. Because it has been shown that a stepwise mutation model with the possibility of large changes in repeat size (by a single mutation) produces genetic diversity in the population that can be approximated by expectations from the infinite allele model of mutations [15], these studies together justify the use of some form of stepwise mutation model for describing the evolutionary properties of STR loci. Of course, the presence of fractional alleles at several of the forensic STR loci [10,11] suggest that there may be multiple processes of mutation simultaneously operating at many of these loci, for which the mathematical description may be similar to the mixed-mutation model proposed by Li [22].

In addition to statistical support for such mutation models, the compilation of observed mutations at the forensic STR loci [12] reveals two further points: 1) the mutation rate does not seem to be strongly dependent upon allele size; and 2) mutations occur more commonly in progenitor alleles, which are more frequent in the population. The latter observation is in direct contradiction with the mutation model (equation 1 on page 59 of the report by Dawid *et al*. [3]), which assumes that the rate of mutation from a progenitor allele (denoted by *i*) is inversely related to its frequency (*π*_*i*_). This, together with the lack of any stationary frequency distributions of alleles labeled by allele sizes, make that mutation model unrealistic and inapplicable to STR loci such as those used in DNA forensics, and probably for other microsatellite loci as well. We should also note that the most widely practiced adjustment of population substructure effects on allelic/genotypic diversity at STR loci [23], as used in our paper [2], is also based on the allele frequency spectra under the balance of mutations and genetic drift, with mutations following the infinite allele model [14]. This approximation is justified by the fact that stepwise occurrences of mutations with a possibility of occasional large size changes yield an allele-frequency spectrum that is nearly identical to that expected under the infinite allele model of mutations [15], and our mutation model allows for such occasional large changes.

We have one last point to make. Although not mentioned by Egeland *et al*. [1], we found an inconsequential error in our original paper [2] while preparing this response. While inferring that equation 8 of our paper [2] represents a true probability distribution (page 7 of that report), we stated '... the summation of equation (8) is always equal to 1 (equation 12)...'. There is in fact no equation (12) in our paper; it should be equation (11). Further the correct representation of equation (11) should be

In conclusion we contend that in absence of any definitive mechanism of mutation models experimentally shown to explain all mutations at STR loci, any mutation model can only be an approximation and hence, the two-phase mutation model described in our paper [2] cannot be readily discounted. In particular, factors that are implicated in the generation of STR mutations include repeat number, repeat motif, length of the repeat unit, flanking sequence, interruptions in the microsatellite, recombination rate, transcription rate, and gender, but not the allele frequency or the number of possible alleles at the locus. The negative relationship between the chance of mutational transitions and frequency of progenitor alleles is directly contradicted by the observation that more mutations are noted for alleles that are more abundant in populations [12]. These data together make the mutation model used by Dawid *et al*. [3] less realistic than the one we used. Thus, the final statement of Egeland *et al*. [1] asserting that our mutation model is not a viable alternative for STR mutations does not have theoretical or empirical support based on the evidence accumulated to date on mutations at such loci.

## Competing interests

The authors declare that they have no competing interests.

## Authors' contributions

RC and JG wrote the manuscript. All authors contributed to the contents, read and approved the manuscript.
